# Transplantation of human endometrial perivascular stem cells with hydroxy saffron yellow A promotes uterine repair in rats

**DOI:** 10.1186/s13287-024-03821-1

**Published:** 2024-07-18

**Authors:** Ning Li, Jialian Mao, Miaomiao Wang, Jiahui Qi, Zhiwei Jiang, Yifan Li, Guijun Yan, Yali Hu, Shiyuan Li, Haixiang Sun, Lijun Ding

**Affiliations:** 1 Center for Reproductive Medicine and Obstetrics and Gynecology, Nanjing Drum Tower Hospital, Clinical College of Nanjing University of Chinese Medicine, Nanjing, 210008 China; 2 Center for Reproductive Medicine and Obstetrics and Gynecology, Nanjing Drum Tower Hospital, Clinical College of Nanjing Medical University, Nanjing, 210008 China; 3 Center for Reproductive Medicine and Obstetrics and Gynecology, Nanjing Drum Tower Hospital, Affiliated Hospital of Medical School, Nanjing University, Nanjing, 210008 China; 4 Department of Vascular Surgery, Nanjing Drum Tower Hospital, Affiliated Hospital of Medical School, Nanjing University, Nanjing, 210008 China; 5 Center for Molecular Reproductive Medicine, Nanjing University, Nanjing, China; 6 Clinical Center for Stem Cell Research, the Affiliated Drum Tower Hospital of Nanjing University Medical School, Nanjing, 210008 China

**Keywords:** Intrauterine adhesion, Hydroxysafflor yellow A, Endometrial perivascular cells, Angiogenesis

## Abstract

**Background:**

Intrauterine adhesions (IUAs) jeopardise uterine function in women, which is a great challenge in the clinic. Previous studies have shown that endometrial perivascular cells (En-PSCs) can improve the healing of scarred uteri and that hydroxysafflor yellow A (HSYA) promotes angiogenesis. The purpose of this study was to observe whether the combination of En-PSCs with HSYA could improve the blood supply and fertility in the rat uterus after full-thickness injury.

**Methods:**

En-PSCs were sorted by flow cytometry, and the effect of HSYA on the proliferation and angiogenesis of the En-PSCs was detected using CCK-8 and tube formation assays. Based on a previously reported rat IUA model, the rat uteri were sham-operated, spontaneously regenerated, or treated with collagen-loaded PBS, collagen-loaded HSYA, collagen-loaded En-PSCs, or collagen-loaded En-PSCs with HSYA, and then collected at both 30 and 90 days postsurgery. HE staining and Masson staining were used to evaluate uterine structure and collagen fibre deposition, and immunohistochemical staining for α-SMA and vWF was used to evaluate myometrial regeneration and neovascularization in each group. A fertility assay was performed to detect the recovery of pregnancy function in each group. RNA-seq was performed to determine the potential mechanism underlying En-PSCs/HSYA treatment. Immunofluorescence, tube formation assays, and Western blot were used to validate the molecular mechanism involved.

**Results:**

The transplantation of Collagen/En-PSCs/HSYA markedly promoted uterine repair in rats with full-thickness injury by reducing fibrosis, increasing endometrial thickness, regenerating myometrium, promoting angiogenesis, and facilitated live births. RNA sequencing results suggested that En-PSCs/HSYA activated the NRG1/ErbB4 signaling pathway. In vitro tube formation experiments revealed that the addition of an ErbB inhibitor diminished the tube formation ability of cocultured En-PSCs and HUVECs. Western blot results further showed that elevated levels of NRG1 and ErbB4 proteins were detected in the Collagen/En-PSCs/HSYA group compared to the Collagen/En-PSCs group. These collective results suggested that the beneficial effects of the transplantation of Collagen/En-PSCs/HSYA might be attributed to the modulation of the NRG1/ErbB4 signaling pathway.

**Conclusions:**

The combination of En-PSCs/HSYA facilitated morphological and functional repair in rats with full-thickness uterine injury and may promote endometrial angiogenesis by regulating the NRG1/ErbB4 signaling pathway.

**Supplementary Information:**

The online version contains supplementary material available at 10.1186/s13287-024-03821-1.

## Background

Intrauterine adhesions (IUAs) are characterised by symptoms such as endometrial fibrosis, menstrual abnormalities, and recurrent miscarriages [1, 2]. The functional layer of the endometrium in normal women undergoes cyclic shedding in response to hormonal changes, and the basal layer plays an important role in promoting functional regeneration [3]. However, intrauterine surgery can cause irreversible damage to the basal layer, leading to impaired regeneration of the functional layer and damaged uterine function. Hysteroscopic adhesion disintegration is the mainstay of clinical treatment for IUA, but the risks of high postoperative recurrence rates and increased likelihood of miscarriage and placental abnormalities remain to be urgently addressed [4, 5]. Alternative therapies such as intrauterine devices, oestrogen supplementation, and amniotic membrane transplantation also have associated drawbacks, such as the occurrence of menstrual abnormalities and decreased pregnancy rates [6–8].

The endometrium, as a unique circulating regenerative tissue in the human body, is intricately tied to vascular growth for functional maintenance [9]. In patients with IUAs, angiogenesis is typically impaired due to the constriction or closure of capillary lumens caused by extensive fibroblast deposits in the stroma [10, 11]. Stem cells have emerged as a promising treatment for IUA, and notable progress has been made in clinical trials [12–14]. Perivascular cells, which originate from the periphery of microvessels, share similar properties with mesenchymal stem cells (MSCs) and exhibit similar immunophenotypes and differentiation potential that are crucial for vascular development and homeostasis maintenance [15, 16]. It has been suggested that vessel walls serve as reservoirs of progenitor cells, with pericytes potentially being the originating fraction of MSCs [17]. Our previous studies revealed that endometrial perivascular cells (En-PSCs) overexpressing angiogenesis inducer 61 (CYR61) regenerated uterine structure and reproductive function in rats with scarred uteri by promoting angiogenesis [18]. En-PSCs were induced to differentiate into vascular endothelial-like cells and uterine stromal-like cells in vitro, which promoted vascular network formation and uterine repair [19].

Hydroxysafflor yellow A (HSYA) is a major active ingredient of safflower, a traditional Chinese medicinal plant that exhibits a broad spectrum of pharmacological activities, particularly in terms of its anti-inflammatory, antioxidant, and cardiovascular effects [20–22]. Previous research has demonstrated that coadministration of HSYA with deferoxamine notably enhances the angiogenic ability of human umbilical vein endothelial cells (HUVECs) and expedites wound healing in diabetic rats by upregulating hypoxia inducible factor-1α (HIF-1α) expression [23]. Additionally, in a vascular dementia model, HSYA upregulated vascular endothelial growth factor (VEGF) expression, leading to increased synaptic plasticity and improved spatial learning and memory in rats [24].

In this study, we explored the reparative effects of coadministration of En-PSCs and HSYA on rats with full-thickness uterine injury and investigated the underlying mechanisms involved. The results suggest that the transplantation of Collagen/En-PSCs/HSYA promotes the restoration of damaged uterine structure and reproductive function in rat uteri with full-thickness injury. The notable promotion of uterine angiogenesis might be related to the activation of the NRG1/ErbB4 signaling pathway.

## Materials and methods

### En-PSCs sorting and culture

En-PSCs were extracted from human endometrium as previously reported [18, 19]. Briefly, endometrial tissues were cut into pieces and digested in 1 mg/mL collagenase I (Sigma, St. Louis, MO, USA) at 37 °C for 30 min, filtered through a sterile 100 μm cell filter, and then lysed by adding 5 times the volume of red blood cell lysis buffer (Beyotime, Shanghai, China) at 4 °C for 15 min, The cells were passed through a 40 μm cell filter and centrifuged, after which the cellular precipitates were resuspended in 1% FBS/PBS, antibodies cocktail containing anti-CD45-APC-Cy7 (1:100; BD Biosciences, San Jose, CA, USA), anti-CD144-PerCPCy5.5 (1:100; BD Biosciences), anti-CD56-PE-Cy7 (1:100; BD Biosciences), anti-CD34-PE (1:100; BD Biosciences), and anti-CD146-FITC (1:100; BD Biosciences) was added to the cell suspensions at 4 °C for 20 min. After washing with 1% FBS/PBS, the CD45-CD144-CD56-CD34-CD146 + endometrial perivascular cells were subsequently sorted on a flow cytometer (BD Biosciences, San Jose, CA, USA). The isolated En-PSCs were cultured in DMEM/F12 (Gibco, Grand Island, NY, USA) supplemented with 10% FBS (Gibco), 1% penicillin–streptomycin (Gibco), and 10 ng/mL bFGF (Gibco) in a 37 °C, 5% CO_2_ cell culture incubator and then passaged when the cells have grown to 80–90% density.

### Flow cytometry analysis

En-PSCs cell surface antigens were detected using flow cytometry (BD Biosciences). The cells were first digested, resuspended in PBS and incubated with CD13-PE (1:100, BD Pharmingen, San Diego, CA, USA), CD29-FITC (1:100, BD Biosciences), CD73-FITC (1:100, BD Biosciences), CD90-PE (1:100, BD Biosciences), CD105-PE (1:100, BD Biosciences), CD146-FITC (1:100, Abclonal, Wuhan, China), CD34-PE (1:100, BD Biosciences), CD45-FITC (1:100, BD Biosciences) and HLA-DR-FITC (1:100, BD Biosciences) for 30 min at room temperature protected from light. After centrifugation and washing, the cells were resuspended in 1% FBS/PBS for detection.

### Multilineage differentiation potential of En-PSCs

The En-PSCs were induced to differentiate into osteogenic, chondrogenic, adipogenic, and neurogenic cells to verify their multidirectional differentiation potential. For osteogenic differentiation, En-PSCs were digested and incubated in 24-well plates at 2 × 10^4^ cells/well. When the cells reached 90% density, the medium was replaced with osteogenic induction medium (Cyagen Orilcell™, Guangzhou, China) to continue the culture. The medium was changed every 3 days, and calcium deposition was observed after 14 days using alizarin red staining. Chondrogenic induction was carried out in 15 mL centrifuge tubes (Corning, NY, USA), En-PSCs were subsequently transferred to centrifuge tubes at a density of 3 × 10^5^, washed and centrifuged in chondrogenic induction medium (Cyagen Orilcell™) to resuspend the cellular precipitates. The incubation process was then continued by placing them vertically in the incubator. After the emergence of the chondrospheres, the bottom of the centrifuge tubes was gently flicked so that the chondrospheres would be suspended within the medium, and the medium was changed every 3 days. After 14 days, the chondrospheres were removed from the tubes, sectioned by paraffin embedding, and stained with alisin blue to observe the effect of chondrogenesis. For adipogenic differentiation, the cells were seeded in 6-well plates at a density of 3 × 10^5^ and differentiation was induced using adipogenic medium (Cyagen Biosciences, Guangzhou, China) when the cells reached 90% confluency. Lipid droplet formation was assessed after 21 days using oil red O staining (Cyagen Biosciences). Prior to the addition of neuron induction medium, preinduction medium supplemented with a concentration of 10^−7^ mol/L all-trans retinoic acid (ATRA; Sigma) was applied for 24 h. Neuro-like differentiation was identified through immunofluorescence staining with neurofilament medium polypeptide (NF-M; 1:100, Santa Cruz Biotechnology, CA, USA) and neuron-specific enolase (NSE; 1:100, Santa Cruz Biotechnology).

### Proliferation efficiency of En-PSCs/HSYA

The effect of different concentrations of HSYA (Shanghai yuanye, Shanghai, China) on the proliferation of En-PSCs was detected using a CCK-8 kit (Fdbio science, Hangzhou, China), En-PSCs (5 × 10^3^/well) were inoculated in 96-well plates, different concentrations of HSYA solution (10 μM, 50 μM, 100 μM, 200 μM, 400 μM, 100 μL solution/well) were added after 24 h, and the control group was complete medium supplemented with 10% FBS. After 24 h of stimulation, 10 μL of CCK-8 solution was added to each well, and the values were analysed by measuring the optical density (OD) at 450 nm using a microplate reader (Thermo, MA, USA) after 4 h of incubation.

### Tube formation assay

Liquid Matrigel (50 μL) (BD Biosciences) was added to 96-well plates at 4 °C and incubated at 37 °C for 30 min, followed by the addition of HUVECs suspended in serum-free F12 medium (2 × 10^4^/well). HUVECs as blank group, 10% FBS complete medium as positive control group, HSYA group (2 × 10^4^ HUVECs), En-PSCs group (1 × 10^4^ En-PSCs/1 × 10^4^ HUVECs), En-PSCs/HSYA group (1 × 10^4^ En-PSCs/1 × 10^4^ HUVECs/50 μM HSYA), incubated at 37 °C for 3 h. Pictures were taken under the microscope. The number of tubes, length (Experimental group length/control group length), and nodes were counted in three wells, with three random fields per well.

Angiogenesis at different ErbB4 levels was observed using a cell co-culture system to validate the results of RNA-seq. Co-culture of En-PSCs with HUVEC (1 × 10^4^ En-PSCs/1 × 10^4^ HUVECs) served as the control group. The concentration of HSYA was 50 μM, and the concentration of ErbB inhibitor (Dacomitinib, MCE, Shanghai, China) was 50 nM [25].

### Transplantation of Collagen/En-PSCs/HSYA for full-thickness injure uterus in rats

Animal experiments were conducted in compliance with the guidelines of Animal Research Ethics Review Committee of the Drum Tower Hospital Affiliated to Nanjing University Medical School and ARRIVE (Animal Research: In Vivo Experiment Report) guidelines 2.0 for approved Laboratory Animal Care and Use. Female 8-week-old SD rats (SiPeiFu, Suzhou, China) were maintained in SPF conditions (Nanjing Drum Tower Hospital Laboratory Animal Center) and allowed to adapt to the new environment. As reported in a previous study [26], the rats weighing 200–250 g with normal 4 d estrous cycle were randomly and uniformly divided into sham-operation group (Sham), spontaneously regenerated group (SR), collagen membrane-loaded PBS group (Collagen/PBS), collagen membrane-loaded HSYA group (Collagen/HSYA), collagen membrane-loaded En-PSCs group (Collagen/En-PSCs), and collagen membrane-loaded En-PSCs/HSYA group (Collagen/En-PSCs/HSYA) (*n* = 8 uterine horns in each group, the experiment was conducted in triplicate. A total of 72 rats were utilized throughout the experiment). After the rats were anesthetized with isoflurane gas (Shenzhen Ruiwode, Shenzhen, China), uterus was cut and exposed bilaterally along the midline of the lower abdomen, an area of the lower and middle uterus approximately 1.5 cm in length and 0.5 cm in width was excised, and the tethered side was preserved. In the Sham group, the uterus was exposed without excision, and in the SR group, the uterus was excised and allowed to heal spontaneously after complete hemostasis. PBS, HSYA, En-PSCs, and En-PSCs/HSYA were in situ delivered onto the uterus with 1.5 cm × 0.5 cm degradable collagen membranes with a 6–0 suture. The abdominal muscular layer was subsequently sutured, and the incision was closed. Twenty units of penicillin were injected per rat to prevent infection for three consecutive days after the operation. The specific process was shown in Fig. S1.

### Histological analysis

At 30 and 90 days post-surgery, rats were euthanized using carbon dioxide inhalation, and their uteruses were collected for histological examination. Uterine injury sites were fixed in 4% paraformaldehyde overnight, dehydrated in gradient ethanol and paraffin embedded. Transverse sections were cut at a thickness of 5 μm. Hematoxylin and eosin (HE) staining was used to visualise the overall structure of the tissue, and endometrial thickness was measured using Image J (National Institutes of Health, Bethesda, MD, USA). Masson staining was used to visualise collagen deposition. Immunohistochemistry was performed using anti-α-smooth muscle actin antibody (α-SMA, 1:2000, Abcam) and anti-von Willebrand factor antibody (vWF, 1:1000, Abcam). Smooth muscle abundance was assessed by the percentage of α-SMA-positive area (α-SMA-positive area of injured area/total α-SMA-positive area). Vessel density was assessed via 40 × microscopy in 3 randomly selected scenes of the injury area. All calculations were performed in Image J.

### Fertility experiments

At 90 days postsurgery, the female rats were cohoused with fertile 12-week-old male SD rats for ten days to evaluate whether the regenerated uterus could accept a fertilised ova and support embryonic development to late gestation. The day of discovery of the vaginal plug was recorded as 0.5 days of pregnancy, and pregnant rats were euthanised at 15.5 days of pregnancy.

### RNA-seq

RNA sequencing analysis was performed using the uterine injury site of rats in the Collagen/En-PSCs group and the Collagen/En-PSCs/HSYA group at 30 days postsurgery (3 vs. 3). Total RNA was extracted with a TRIzol kit (Thermo Fisher Scientific, Rockford, IL, USA), after which the RNA purity and quality were determined with a NanoDrop spectrophotometer. RNA integrity was assessed by an Agilent 2100 Bioanalyzer. A TruSeq Stranded mRNA LT Sample Prep Kit (Illumina, San Diego, CA, USA) was used to construct the libraries. RNA sequencing was performed by Shanghai Ouyi Biotechnology Co, Ltd (China). The library was sequenced on the Illumina platform, and the raw sequencing data were subjected to FastQC quality control to remove low-quality reads. The resulting CleanReads were subsequently aligned with the reference genome, and the DEGs were analysed via DESeq2 between the two groups. The results were subjected to gene enrichment analysis via Gene Ontology (GO) and Kyoto Encyclopedia of Genes and Genomes (KEGG) enrichment analyses.

### Immunofluorescence analysis

Immunofluorescence staining was performed to detect NRG1 and ErbB4 expression in En-PSCs and HUVECs. En-PSCs and HUVECs were added to the chamber slide (Thermo Fisher Scientific) at a volume of 2 × 10^3^/200 μL per well, washed twice in PBS after 24 h, and fixed in 4% paraformaldehyde for 10 min at room temperature. Anti-NRG1 (1:100, Abclonal) and anti-ErbB4 (1:100, Santa Cruz Biotechnology) were used as the primary antibodies overnight at 4 °C. The second day was incubated with secondary Alexa Fluor 594-conjugated goat anti-mouse IgG (1:500, Invitrogen, Grand Island, NY, USA) and Alexa Fluor 488-conjugated goat anti-rabbit IgG (1:500, Invitrogen) at room temperature for 1 h, and then the nuclei were stained with DAPI (Sigma). The cells were imaged by fluorescence confocal microscopy (Leica, Wetzlar, Germany).

### Western blot

Uterine samples were collected 30 days after surgery, and tissue proteins were extracted using RIPA lysis buffer (epizyme, Shanghai, China) supplemented with protease inhibitors (Beyotime). After BCA (Beyotime) quantification, protein blot was carried out using an equivalent 40 μg volume of total protein. After separated from the SDS gel, the proteins were transferred to a PVDF membrane (Millipore, Bedford, MA, USA). 5% skim milk was used for blocking at room temperature for 1.5 h, and the membranes were incubated with primary antibodies against NRG1 (1:1000, Abclonal), ErbB4 (1:500, Santa Cruz Biotechnology), or GAPDH (1:10,000, Bioworld, St Louis Park, MN, USA) overnight at 4 °C. Goat Anti-Rabbit IgG (Abways, Shanghai, China) was used as the secondary antibody at room temperature for 1 h. The bands were visualised using an enhanced chemiluminescence (ECL) kit (Beyotime).

### Statistical analyses

The data were analysed using one-way ANOVA for multiple group comparisons and are expressed as the mean ± SD. Pregnancy rates are expressed as counts and percentages. Significant differences were calculated using GraphPad Prism version 9 (GraphPad Software, La Jolla, USA). A difference was considered to be statistically significant when the *P* value was < 0.05.

## Results

### En-PSCs/HSYA promote angiogenesis in vitro

Flow cytometric analysis revealed that the En-PSCs expressed CD13 (99.3%), CD29 (99.3%), CD73 (99.5%), CD90 (99.5%), CD105 (99.6%), and CD146 (90%), but had low expression of CD34 (0.22%), CD45 (0%), and HLA-DR (0.5%) (Fig. S2A), which reached the criteria set by the Mesenchymal and Tissue Stem Cell Committee of the International Society for Cellular Therapy for defining human MSCs [27], and its findings were consistent with those of previous studies [17, 18]. In addition, after in vitro cultured in osteogenic, chondrogenic, adipogenic, and neurogenic induction medium, calcium deposition was detected through alizarin red staining (Fig. S2B), and the presence of endoacidic mucopolysaccharides in cartilage tissues was detected by alizarin blue staining (Fig. S2C). Lipid droplet formation was detected by oil red O staining (Fig. S2D), and neural-like differentiation was confirmed by immunofluorescence staining for NF-M and NSE (Fig. S2E, F). These results suggested that En-PSCs had multilineage differentiation potential.

**Fig. 1:**
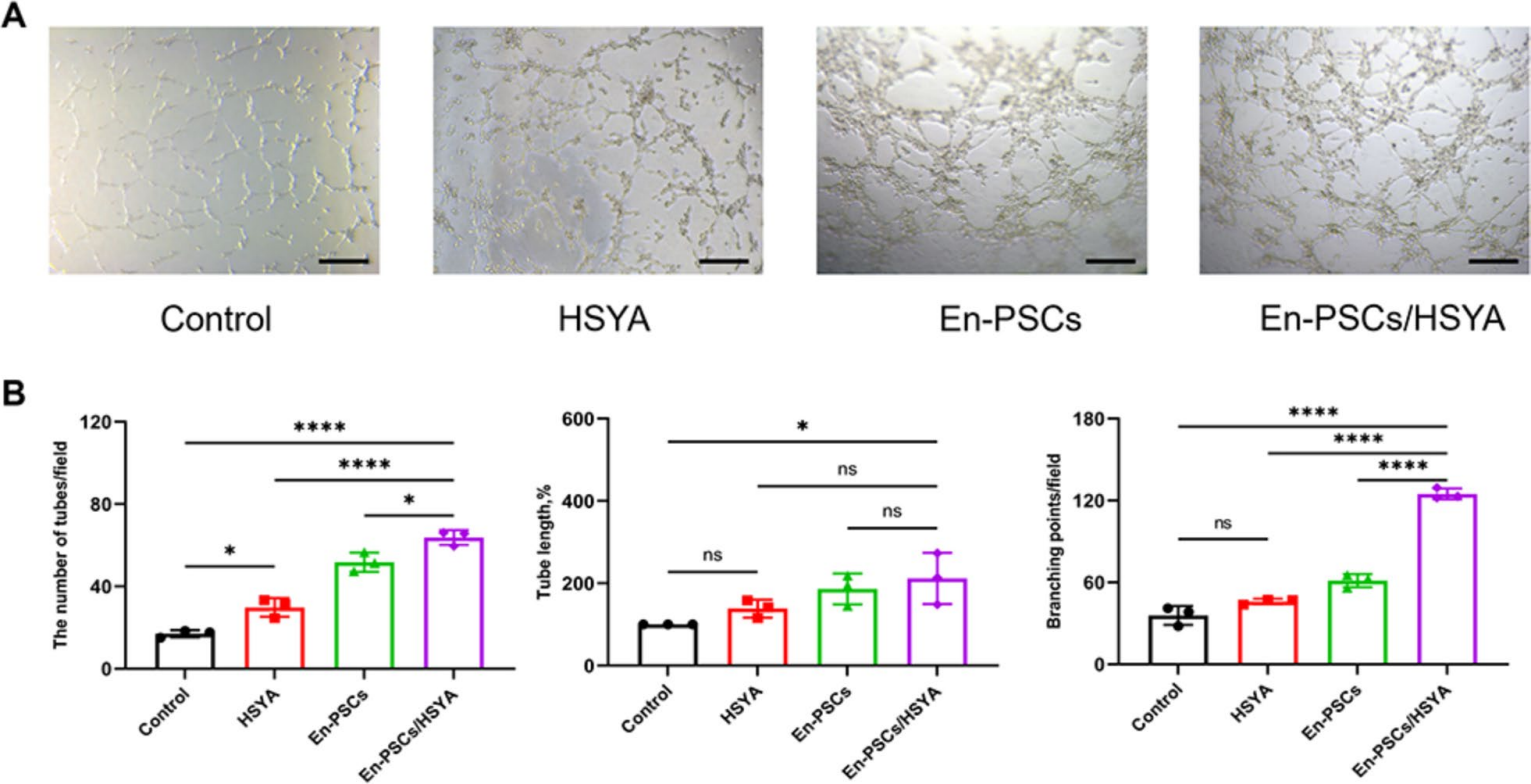
En-PSCs/HSYA induced angiogenesis. **A** Tube formation in the positive control group, HSYA group, En-PSCs group and En-PSCs/HSYA group under a microscope. Scale bar, 500 µm. **B** Statistical analysis of the number of tubes, the tube length, and the number of branching points in each group. The experiment was conducted in triplicate for each group. Data are expressed as mean ± SD. *P < 0.05, **P < 0.01, ***P < 0.001, ****P < 0.0001, *ns* Not significant

As shown in Fig. S3, the proliferation of the En-PSCs cultured with 50 μM HSYA was comparable to that of the control cells under normal culture conditions. The angiogenic capacity of HSYA was assessed by a tube formation assay **(**Fig. 1A**)**. The number of tubes in the HSYA-treated HUVECs (29.780 ± 4.538) was greater than that in the control group (17.000 ± 1.764, *P* < 0.05). The length and number of nodes were not significantly different, although they tended to increase, indicating that HSYA had a positive effect on promoting angiogenesis. The En-PSCs/HSYA group exhibited a higher number of tubes (63.780 ± 3.564) and nodes (124.800 ± 3.977) compared to the En-PSCs group (51.780 ± 4.683, *P* < 0.05; 61.330 ± 4.807, *P* < 0.0001) **(**Fig. 1B**)**. The results indicated that the application of En-PSCs/HSYA demonstrated a superior capacity in promoting angiogenesis compared to En-PSCs alone.

### Transplantation of Collagen/En-PSCs/HSYA prompts endometrial regeneration

**Fig. 2:**
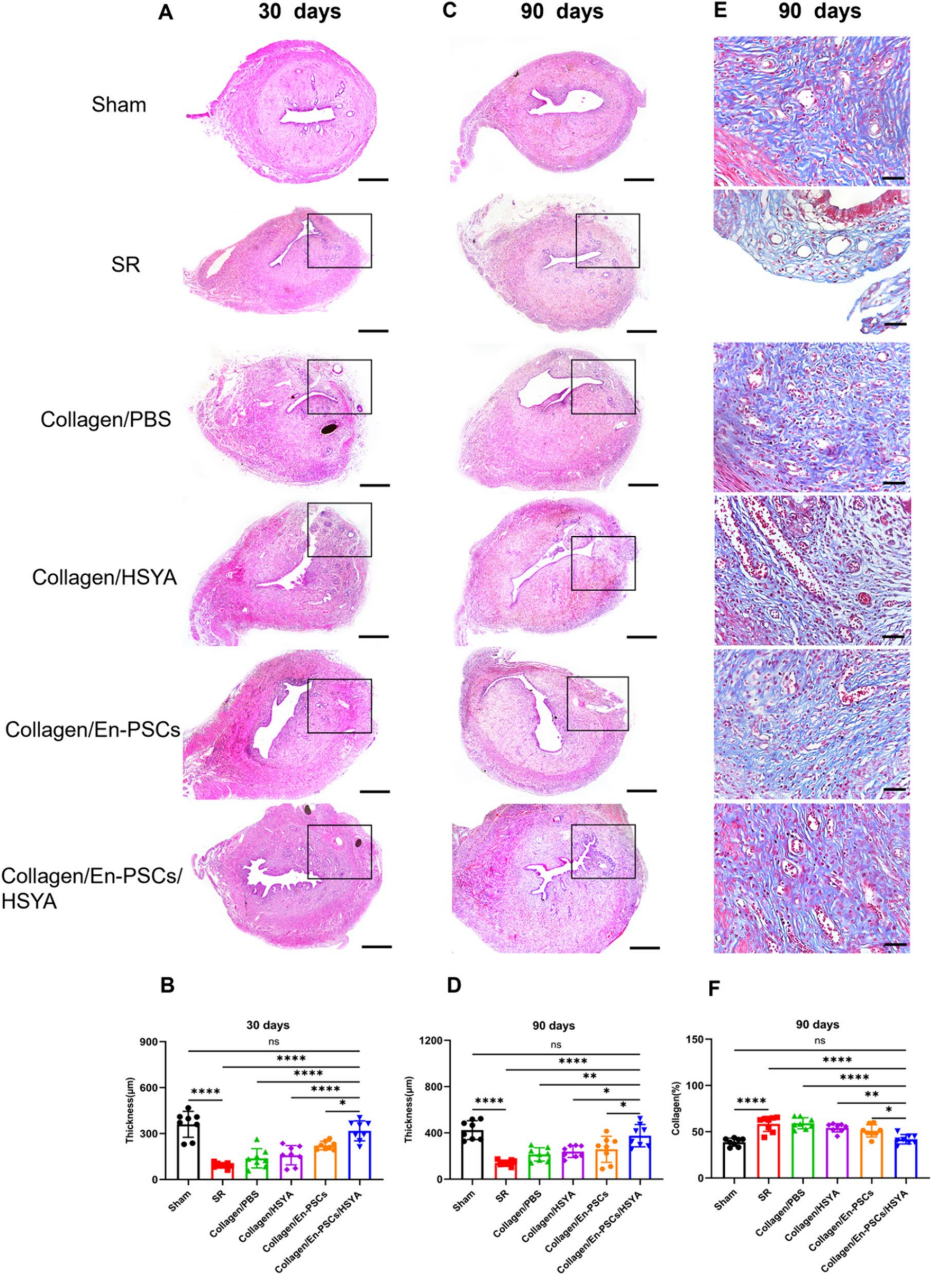
Morphological analysis of the uterus and degree of fibrosis after the transplantation of Collagen/En-PSCs/HSYA. **A** Structural depiction of H&E staining in each group at 30 days postsurgery and **B** statistical analysis of endometrial thickness, with damaged areas on the black border. Scale bar, 500 µm. **C** Structural image of H&E-stained sections from each group at 90 days postsurgery and **D** statistical analysis of endometrial thickness at 90 days, with damaged areas on the black border. Scale bar, 500 µm. **E** Masson staining results for each group at 90 days postsurgery. **F** Statistical analysis of Masson staining in each group at 90 days postsurgery. The experiment was conducted in triplicate for each group (n = 8 uteri per group). Data are expressed as mean ± SD. *P < 0.05, **P < 0.01, ***P < 0.001, ****P < 0.0001, *ns* Not significant

All histological sections were assessed by three independent observers (Author 1, 9 and 11) who were blinded to the groups. Rat uterine tissues were collected 30 days after full-thickness injury, and the endometrial thickness and the number of glands in the damaged area were assessed using haematoxylin–eosin (HE) staining. The staining results revealed significantly improved structural integrity of the uterine cavity and an increased number of glands after the transplantation of Collagen/En-PSCs/HSYA (Fig. 2A). The thickness of the endometrium and the number of glands in the Collagen/En-PSCs/HSYA group (317.800 ± 65.260 μm; 27.000 ± 8.142) were significantly greater than those in the SR (92.690 ± 17.660 μm, *P* < 0.0001; 12.630 ± 3.462, *P* < 0.01), Collagen/PBS (138.500 ± 62.880 μm, *P* < 0.0001; 16.000 ± 8.350, *P* < 0.05), Collagen/HSYA (158.600 ± 62.700 μm, *P* < 0.0001; 15.630 ± 7.070, *P* < 0.05), and Collagen/En-PSCs groups (221.600 ± 27.900 μm, *P* < 0.05; 17.880 ± 5.718, ns) (Fig. 2B) (Fig. S4A). At 90 days postsurgery, the uterine cavity of the rats in the Collagen/En-PSCs/HSYA group was structurally intact (Fig. 2C), with the endometrial thickness (375.800 ± 96.520 μm) surpassing that of the rats in the SR group (140.700 ± 23.650 μm, *P* < 0.0001), the Collagen/PBS group (214.100 ± 57.590 μm, *P* < 0.01), the Collagen/HSYA group (238.300 ± 50.880 μm, *P* < 0.05), and the Collagen/En-PSCs group (258.300 ± 112.400 μm, *P* < 0.05) (Fig. 2D).The number of glands in the Collagen/En-PSCs/HSYA group (31.380 ± 4.406) was considerable to the Sham group (31.250 ± 8.031) (Fig. S4B).

Given that fibrosis is one of the pathological features of IUA, Masson staining was used to detect collagen deposition and the degree of fibrosis in each group (Fig. 2E). Transplantation of the Collagen/En-PSCs/HSYA (41.820% ± 5.062%) reduced the degree of fibrosis, as indicated by a decreased area of collagen staining compared with that in the SR group (58.440% ± 8.163%, *P* < 0.0001), the Collagen/PBS group (59.160% ± 6.016%, *P* < 0.0001), the Collagen/HSYA group (54.230% ± 4.497%, *P* < 0.01), and the Collagen/En-PSCs group (50.920% ± 6.643%, *P* < 0.05) (Fig. 2F). The results suggested that treatment with Collagen/En-PSCs/HSYA reduced fibrosis in the injured area and enhanced the structural recovery of the uterus.

### Transplantation of Collagen/En-PSCs/HSYA induces myometrial reconstruction and angiogenesis

**Fig. 3:**
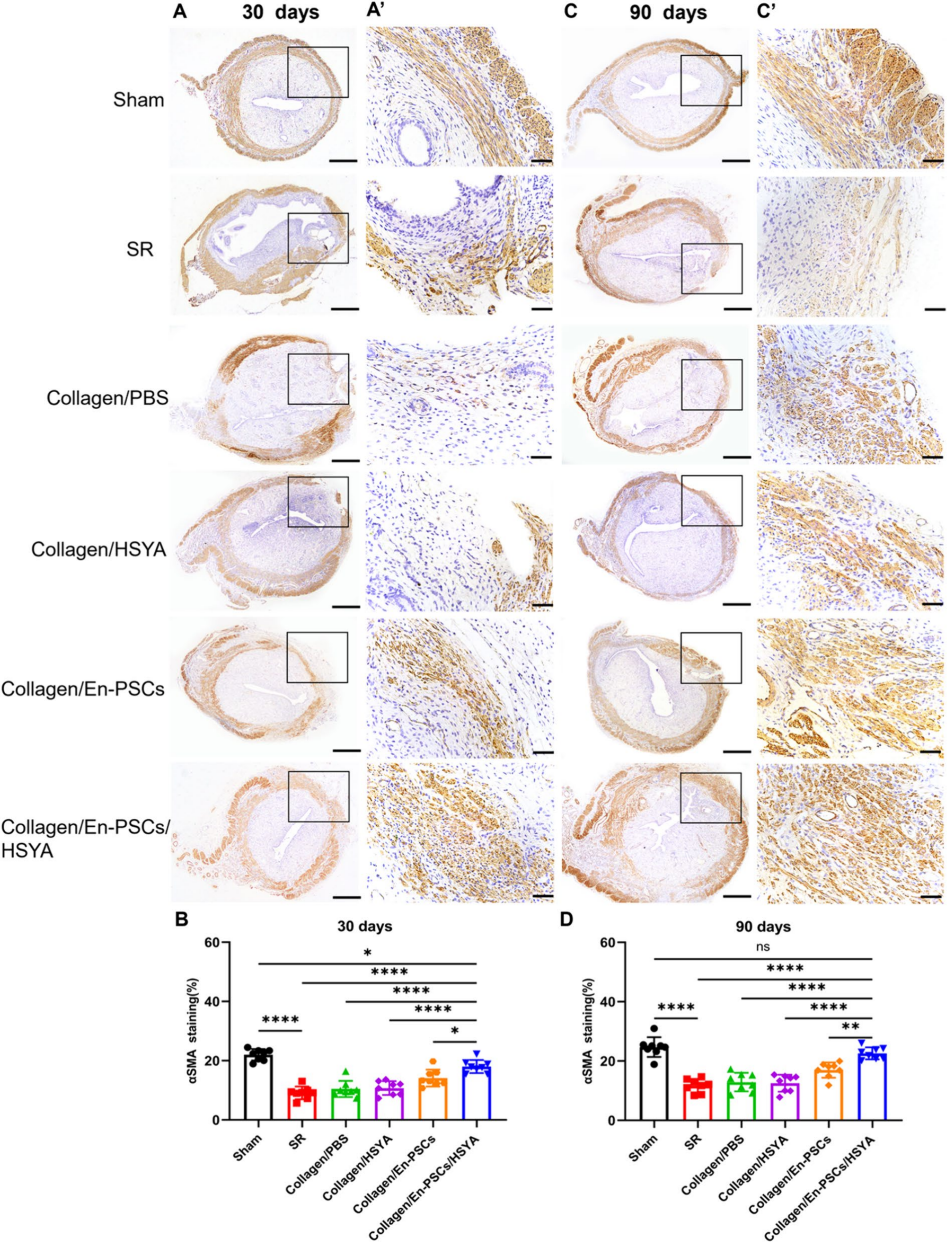
Immunohistochemical staining for α-SMA after the transplantation of Collagen/En-PSCs/HSYA. **A** Structural image of α-SMA staining in each group at 30 days postsurgery and **B** statistical analysis of the proportion of the α-SMA-positive area to the total uterine area. Scale bar, 500 µm. The black border delineates the damaged area. A local magnification of Figure **A** is shown in Figure **A’**. Scale bar, 50 μm. **C** Structural image of α-SMA staining in each group at 90 days postsurgery. Scale bar, 500 µm. **D** Statistical analysis of the proportion of the positivity. Local magnification of Figure **C** is shown in Figure **C’**. Scale bar, 50 μm. The experiment was conducted in triplicate for each group (*n* = 8 uteri per group). Data are expressed as mean ± SD. *P < 0.05, **P < 0.01, ***P < 0.001, ****P < 0.0001, *ns* Not significant

Muscle regeneration in the damaged area of the uterus was detected using α-SMA staining at 30 days postsurgery (Figs. 3A, A**’**). Significant smooth muscle bundle regeneration was observed after treatment with Collagen/En-PSCs/HSYA, in which the α-SMA-positive ratio (18.010% ± 2.195%) exceeded that in the SR group (9.117% ± 2.136%, *P* < 0.0001), the Collagen/PBS group (10.450% ± 2.733%, *P* < 0.0001), the Collagen/HSYA group (10.730% ± 2.324%, *P* < 0.0001), and the Collagen/En-PSCs group (14.150% ± 2.847%, P < 0.05) (Fig. 3B). At 90 days postsurgery (Fig. 3C, C**’**), the Collagen/En-PSCs/HSYA group exhibited regularly arranged myofibers, and the α-SMA-positive ratio (22.590% ± 2.026%) was greater than that in the SR group (11.700% ± 2.266%, P < 0.0001), the Collagen/PBS group (12.900% ± 3.124%, *P* < 0.0001), the Collagen/HSYA group (12.540% ± 2.848%, *P* < 0.0001), and the Collagen/En-PSCs group (16.970% ± 2.569%, *P* < 0.01) (Fig. 3D).

**Fig. 4:**
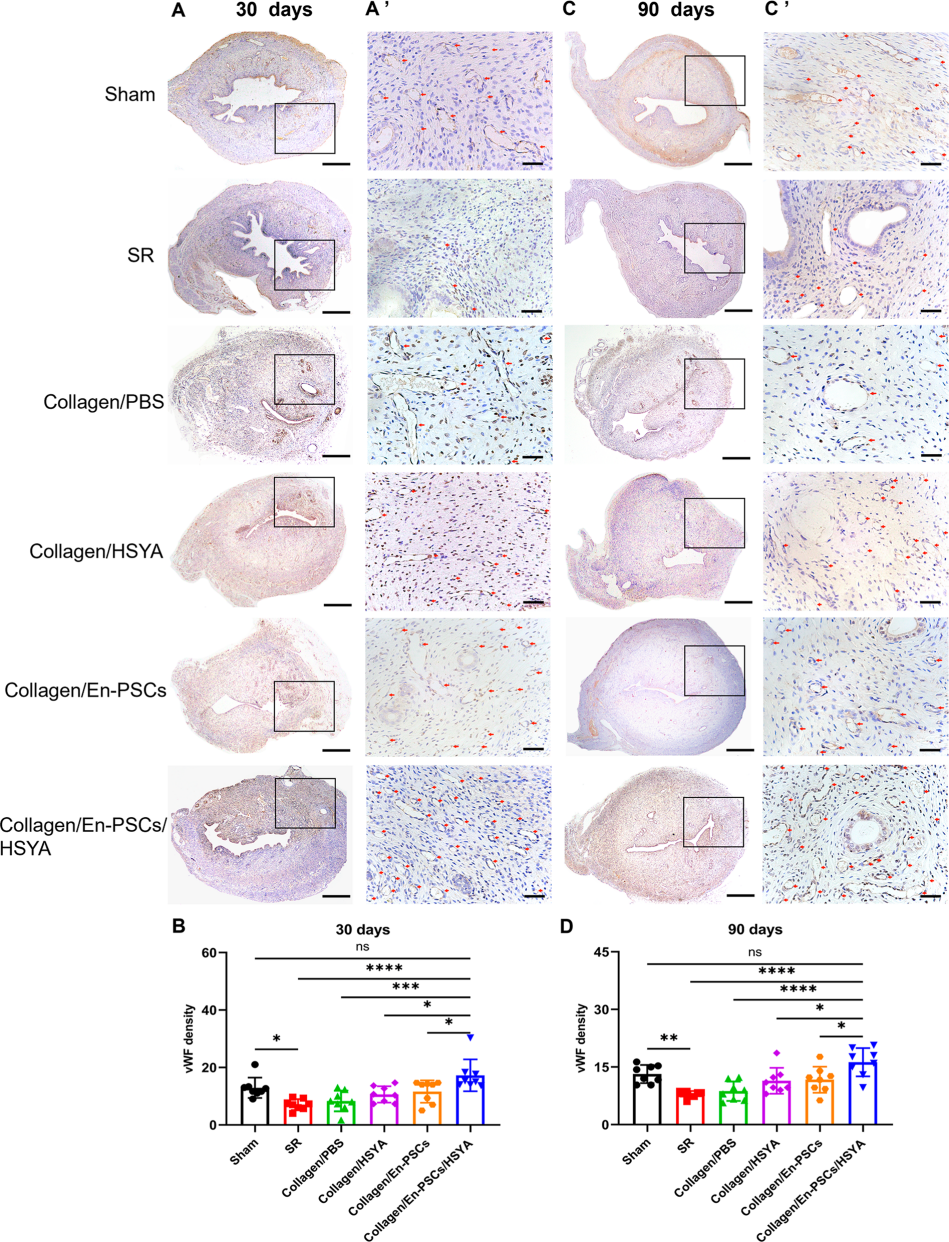
Immunohistochemical staining for vWF after the transplantation of Collagen/En-PSCs/HSYA. **A** Graphs of vWF staining in each group at 30 days postsurgery and **B** the corresponding quantitative statistical graph. Scale bar, 500 µm. The black border marks the damaged sites. Local magnifications are presented in **A’**. Scale bar, 50 µm. **C** Graph of the immunohistochemical vWF staining results for each group at 90 days postsurgery and **D** the statistical analysis of vascular density. Scale bar, 500 µm. The black border marks the damaged sites. Local magnifications are presented in **C’**. Scale bar, 50 µm. The experiment was conducted in triplicate for each group (*n* = 8 uteri per group). Data are expressed as mean ± SD. *P < 0.05, **P < 0.01, ***P < 0.001, ****P < 0.0001, ns, not significant

To evaluate angiogenesis in the damaged region of the uterus, microvessel density was measured using vWF staining. At 30 days posttransplantation, there was a significant increase in the number of neovessels in the damaged area in the Collagen/En-PSCs/HSYA group (17.290 ± 5.545) (Figs. 4A, A**’**), which exhibited a more homogeneous distribution of blood vessels and greater microvessel density than did the SR group (7.083 ± 1.884, *P* < 0.0001), the Collagen/PBS group (8.333 ± 3.617, *P* < 0.001), the Collagen/HYSA group (10.630 ± 2.848, *P* < 0.05), and the Collagen/En-PSCs group (11.630 ± 3.922, *P* < 0.05) (Fig. 4B). After 90 days (Fig. 4C, C**’**), the number of neovessels in the Collagen/En-PSCs/HSYA group (16.250 ± 3.668) was significantly greater than that in the SR group (7.833 ± 0.909, *P* < 0.0001), the Collagen/PBS group (8.708 ± 2.523, *P* < 0.0001), the Collagen/HSYA group (11.420 ± 3.375, *P* < 0.05), and the Collagen/En-PSCs group (11.710 ± 3.397, *P* < 0.05) (Fig. 4D).

### Transplantation of Collagen/En-PSCs/HSYA restores fertility

**Fig. 5:**
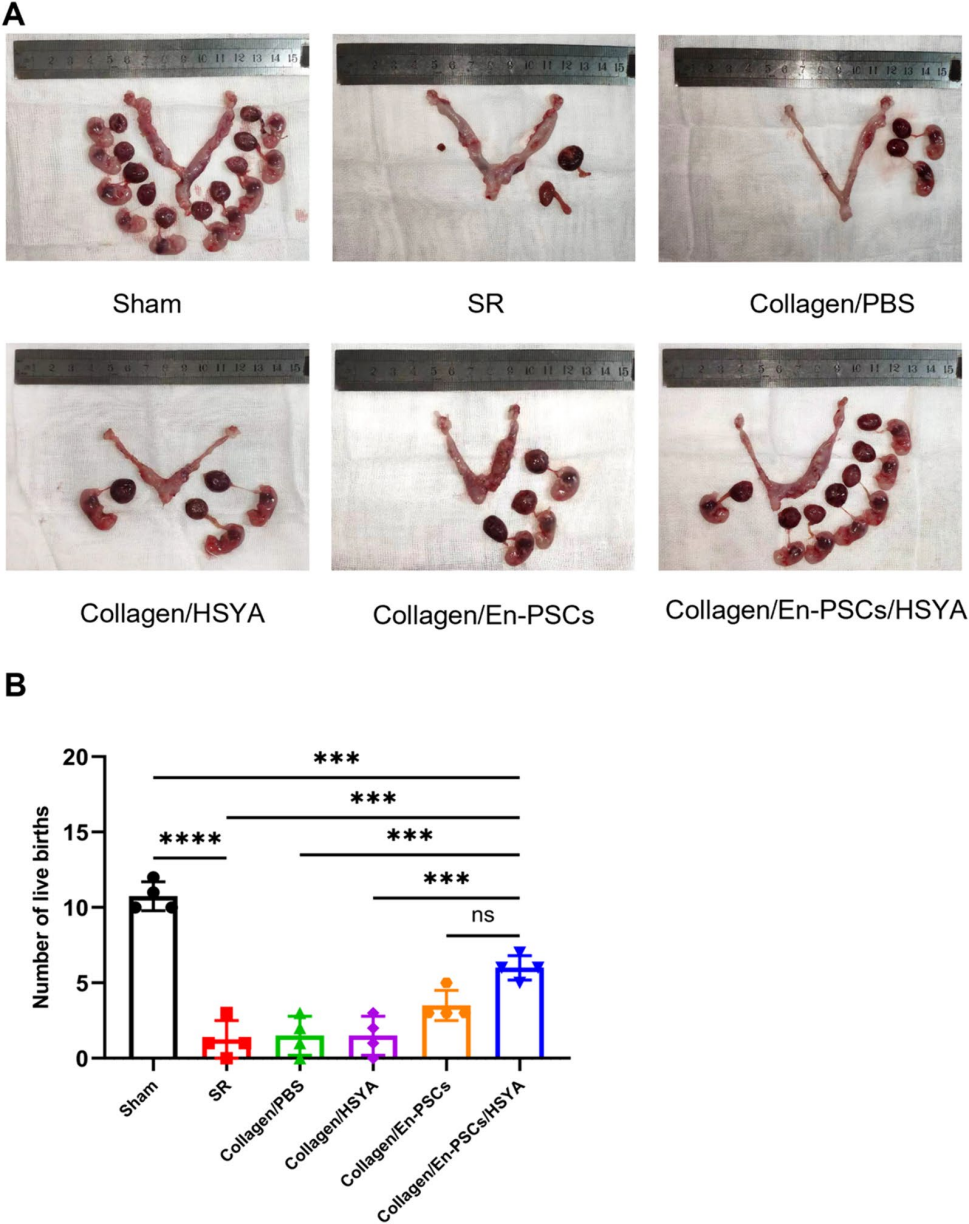
Recovery of fertility function in rats after the transplantation of Collagen/En-PSCs/HSYA. **A** Results of the fertility test in each group at 90 days after surgery. **B** The number of live fetuses at late gestation in each group (*n* = 8 uteri per group). Data are expressed as mean ± SD. *P < 0.05, **P < 0.01, ***P < 0.001, ****P < 0.0001, *ns* Not significant

The uterus, a key reproductive organ, has the capacity to support embryo implantation and development, and it serves as an intuitive indicator of uterine function recovery. The outcomes of the fertility experiment demonstrated a noteworthy increase in the number of embryos and embryonic development in the Collagen/En-PSCs/HSYA group (6.000 ± 0.817), which was significantly higher than that in the SR group (1.250 ± 1.258, *P* < 0.001), Collagen/PBS group (1.500 ± 1.291, *P* < 0.001), and Collagen/HSYA group (1.500 ± 1.291, *P* < 0.001). However, there was no statistically significant difference compared to the Collagen/En-PSCs group, but a noticeable increasing trend was observed instead. These findings indicate that transplantation of Collagen/En-PSCs/HSYA has improved the fertility of rats with intrauterine adhesions (Fig. 5A, B) (Table 1).

**Table 1:** Fertility outcomes in each group at 90 days postoperatively

Clusters	Total number of uteri	The number of pregnant uteri (%)	The number of uteri with embryo implantation at the injured site (%)
Sham	8	8(100)	
SR	8	4(50)	1(12.5)
Collagen/PBS	8	4(50)	2(25)
Collagen/HSYA	8	4(50)	2(25)
Collagen/En-PSCs	8	5(62.5)	5(62.5)
Collagen/En-PSCs/ HSYA	8	6(75)	6(75)

### Collagen/En-PSCs/HSYA improve uterine regeneration through the NRG1/ErbB4 pathway

To elucidate the effect of HSYA on En-PSCs, RNA sequencing analysis was performed on six regenerated rat uterus samples (from the three En-PSCs-treated groups and three En-PSCs/HSYA-treated Groups 30 days after surgery) to explore the molecular mechanism by which the En-PSCs/HSYA system promotes regeneration of the injured uterus. The RNA sequencing results revealed 134 upregulated genes and 436 downregulated genes after En-PSCs/HSYA supplementation compared to those in the En-PSCs group (Fig. S5A, B). Based on these differentially expressed genes (DEGs), GO enrichment analysis revealed that the DEGs were enriched mainly in hormone regulation and neurogenesis (Fig. S5C). KEGG analysis revealed that the ErbB, EGFR, and cAMP signaling pathways potentially contributed to the effect of the En-PSCs/HSYA treatment (Fig. S5D).

ErbB receptor deficiency reportedly leads to embryonic death at implantation, multiorgan epithelial underdevelopment, and organ failure [28, 29]. ErbB4 overexpression has also shown promise in improving the phenotype of senescent MSCs, enhancing vascular density, and ameliorating cardiac function in infarcted mice [30].

**Fig. 6:**
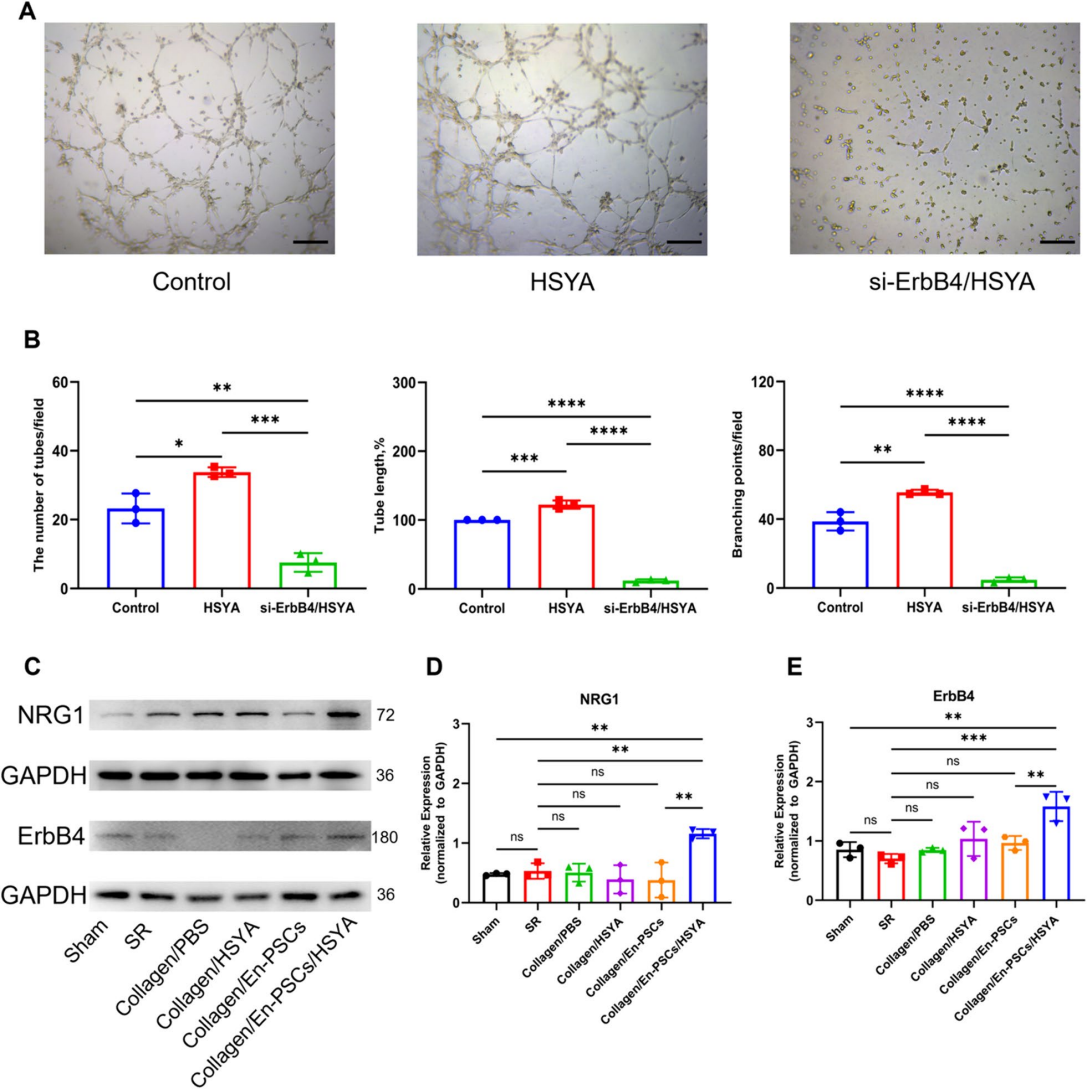
The NRG1/ErbB4 interaction enhances angiogenesis after treatment with En-PSCs/HSYA. **A** Tube assays of the control, HSYA and HSYA/si-ErbB4 groups under a microscope. Scale bar, 500 μm. **B** Statistics of the number of tubes, tube length, and number of branching points in each group, with three random scenes taken in each group. **C** Western blot analysis of NRG1 and ErbB4 expression in each group. **D** NRG1/GAPDH densitometric analysis of each group (*n* = 3 uteri per group). **E** ErbB4/GAPDH densitometric analysis of each group (*n* = 3 uteri per group). The experiment was conducted in triplicate for each group. Data are expressed as mean ± SD. *P < 0.05, **P < 0.01, ***P < 0.001, ****P < 0.0001, *ns* Not significant

Immunofluorescence revealed that both the En-PSCs and HUVECs expressed NRG1 and ErbB4 (Fig. S6), which indicated that angiogenesis was possibly affected by receptor–ligand interactions. As shown in Fig. 6A, in the in vitro tube formation assays, the number of tubes (33.780 ± 1.388), tube length (122.300% ± 6.090%) and number of nodes (55.560 ± 1.540) were greater in the HSYA-treated group than in the control group (23.220 ± 4.338, *P* < 0.05; 100% ± 0%, *P* < 0.001; 38.670 ± 5.333, *P* < 0.01). Conversely, the tube-forming ability was not improved by HSYA treatment after ErbB4 was downregulated in the si-ErbB4/HSYA group (7.556 ± 2.694, *P* < 0.001; 12.280% ± 1.888, *P* < 0.0001; 4.889 ± 1.388, *P* < 0.0001) **(**Fig. 6B**)**. Therefore, the NRG1/ErbB4 signaling pathway may be responsible for the effect of HSYA on tube formation in En-PSCs.

Next, the NRG1 and ErbB4 protein levels in the regenerated uterus were detected via Western blot. As shown in Fig. 6C, NRG1 and ErbB4 protein levels were increased in the Collagen/En-PSCs/HSYA group compared with those in the Collagen/En-PSCs group (Figs. 6D, E), suggesting that Collagen/En-PSCs/HSYA may promote uterine repair by regulating the NRG1/ErbB4 signaling pathway. Full-length blots are presented in Fig. S7.

## Discussion

In the present study, we demonstrated that the combined application of Collagen/En-PSCs/HSYA can effectively restore uterine structure and reproductive function by promoting angiogenesis in the damaged uterus.

Angiogenesis influences the remodelling and maturation of the vascular network, playing a pivotal role in endometrial repair and regeneration, which, in turn, has a significant impact on embryo implantation [31]. Pericytes residing in the pericapillary area play a crucial role in vascular development, stabilisation, and remodelling [32]. En-PSCs express mesenchymal stem cell markers, possess multispectral differentiation capacity, and exhibit immunomodulatory potential [33]. Previous studies have indicated the positive reparative effects of En-PSCs in a full-thickness injured uterus in a rat IUA model [18]. HSYA is a monomeric compound extracted from the medicinal plant safflower that is widely used in ischaemic organs requiring a rich blood supply, such as the brain and heart [34, 35]. Previous studies have shown that HSYA promotes HUVECs migration and angiogenesis by modulating angiopoietin-1 (Ang-1) expression, leading to an increased number of neovessels in the ischaemic gastrocnemius muscle [36]. Furthermore, HSYA improves endothelial cell hypoxia tolerance and promotes cell proliferation under hypoxic conditions by enhancing VEGF expression and increasing HIF-1α transcriptional activity [37]. In the present study, we found that the combination of En-PSCs/HSYA was more effective than the En-PSCs alone at promoting HUVEC tube formation. These results were further validated via in vivo experiments, in which compared with Collagen/En-PSCs, Collagen/En-PSCs/HSYA induced an increase in vWF-positive neovessels, promoting the repair and regeneration of the damaged endometrium.

Neuregulin-1 (NRG-1), a member of the epidermal growth factor (EGF) family, plays a crucial role in promoting the development of the cardiovascular system and the nervous system [38]. NRG1 is a ligand for the ErbB family of receptor tyrosine kinases. The binding of NRG1 to the receptor results in the formation of homodimers or heterodimers, triggering the activation of the signaling cascade that influences the dynamics of cell migration and proliferation and the repair response in response to organismal injury in a paracrine manner [39, 40]. Previous studies have shown that NRG1 promotes nitric oxide (NO) production through the activation of endothelial nitric oxide synthase (eNOS) in cardiomyocytes, which is known to promote endothelial cell proliferation and angiogenesis [41, 42]. Exogenous supplementation of NRG1 has been shown to promote blood flow recovery and arteriogenesis in hindlimb ischaemic mice [43]. In the present study, the immunofluorescence results showed that both En-PSCs and HUVECs expressed NRG1 and ErbB4. An in vitro tube formation assay suggested that HSYA promotes the cotubulation of En-PSCs/HUVECs, which was inhibited by the addition of an ErbB receptor inhibitor, indicating the involvement of NRG1/ErbB4 interactions in enhancing the angiogenic capacity of En-PSCs/HUVECs in the presence of HSYA. In the in vivo study, compared with rats in other treatment groups, rats treated with the combination of En-PSCs/HSYA exhibited superior endometrial angiogenesis, and the contributing role of NRG1/ErbB4 activation was also confirmed by protein level. Taken together, these findings suggest that the combination of En-PSCs and HSYA can decrease the degree of endometrial fibrosis, promote myometrial reconstruction, and increase angiogenesis by regulating NRG1-ErbB4 ligand receptor binding, thereby facilitating the repair of the damaged uterus.

In terms of clinical efficacy, the combined treatment presented advantages in promoting uterine angiogenesis and enhancing uterine function compared to conventional hysteroscopic adhesiolysis. Our previous studies have shown that a collagen scaffold with autologous bone marrow mononuclear cells improved uterine morphology, blood supply, and menstrual status in patients with severe IUA. Five patients in this study ultimately gave birth to healthy babies [44]. In a subsequent randomized controlled clinical trial involving 140 patients with moderate to severe IUA, 62.5% (45/72) of the patients transplanted with a collagen membrane combined with autologous bone marrow stem cells achieved successful pregnancy, which was significantly higher than the rate in the control group (28/68, 41.2%) [45]. These findings suggested that the binding of materials to stem cells can effectively improve uterine structure, restore fertility, and ensure safety. The endometrial perivascular stem cells (En-PSCs) utilized in this study exhibited a high degree of homology to uterine tissue and showed superior proliferation capacity when compared to bone marrow stem cells [46]. As a natural compound, HSYA has a positive impact on promoting angiogenesis. Serving as a delivery carrier, the collagen membrane is highly biocompatible and can be safely degraded in vivo [44]. Its porous structure provides space and oxygen for cell growth, enabling cells to effectively interact with the damaged site, promoting cell growth and proliferation, ultimately improving therapeutic efficiency [26]. The effectiveness of Collagen/En-PSCs/HSYA has been demonstrated in this preclinical study, and the specific clinical impact is worth further investigation.

In this study, we reported the combination of Collagen/En-PSCs/HSYA for the treatment of severe uterine damage for the first time. The observed effects may be linked to the NRG1-ErbB4 signaling pathway. However, further studies are necessary to elucidate the downstream regulatory mechanisms involved.

## Conclusion

Collagen/En-PSCs/HSYA transplantation promoted endometrial regeneration, myometrial repair and angiogenesis in full-thickness injured uterus of rats and restored fertility, and the underlying mechanism may be related to the modulation of the NRG1/ErbB4 signaling pathway. However, further studies are needed to investigate the clinical value of this system for IUA patients.

### Supplementary Information

Supplementary file 1.

## Data Availability

The RNA-seq data used in this study have been deposited in NCBI Short Read Archive (SRA) under the project number PRJNA1123859. Please access the link for https://www.ncbi.nlm.nih.gov/bioproject/PRJNA1123859.
