# Supplementary material for: Transplantation of human endometrial perivascular stem cells with hydroxy saffron yellow A promotes uterine repair in rats
Source: Stem Cell Res Ther. 2024 Jul 18;15:217. doi: 10.1186/s13287-024-03821-1 (PMC11256499; doi:10.1186/s13287-024-03821-1)
Supplement: Supplementary file 1 [file 13287_2024_3821_MOESM1_ESM.docx]

**Additional files**


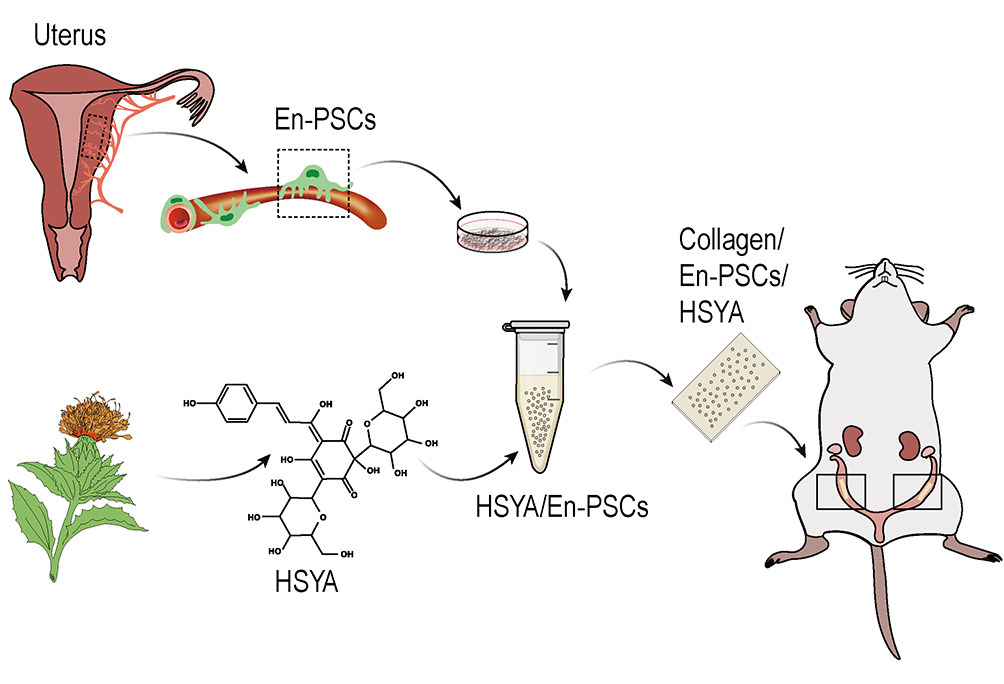


**Fig. S1 Flow chart of the systematic approach utilising a collagen membrane-based coculture system for the *in situ* delivery of HSYA and En-PSCs, emphasising targeted repair and functional regeneration in the rat IUA model.**


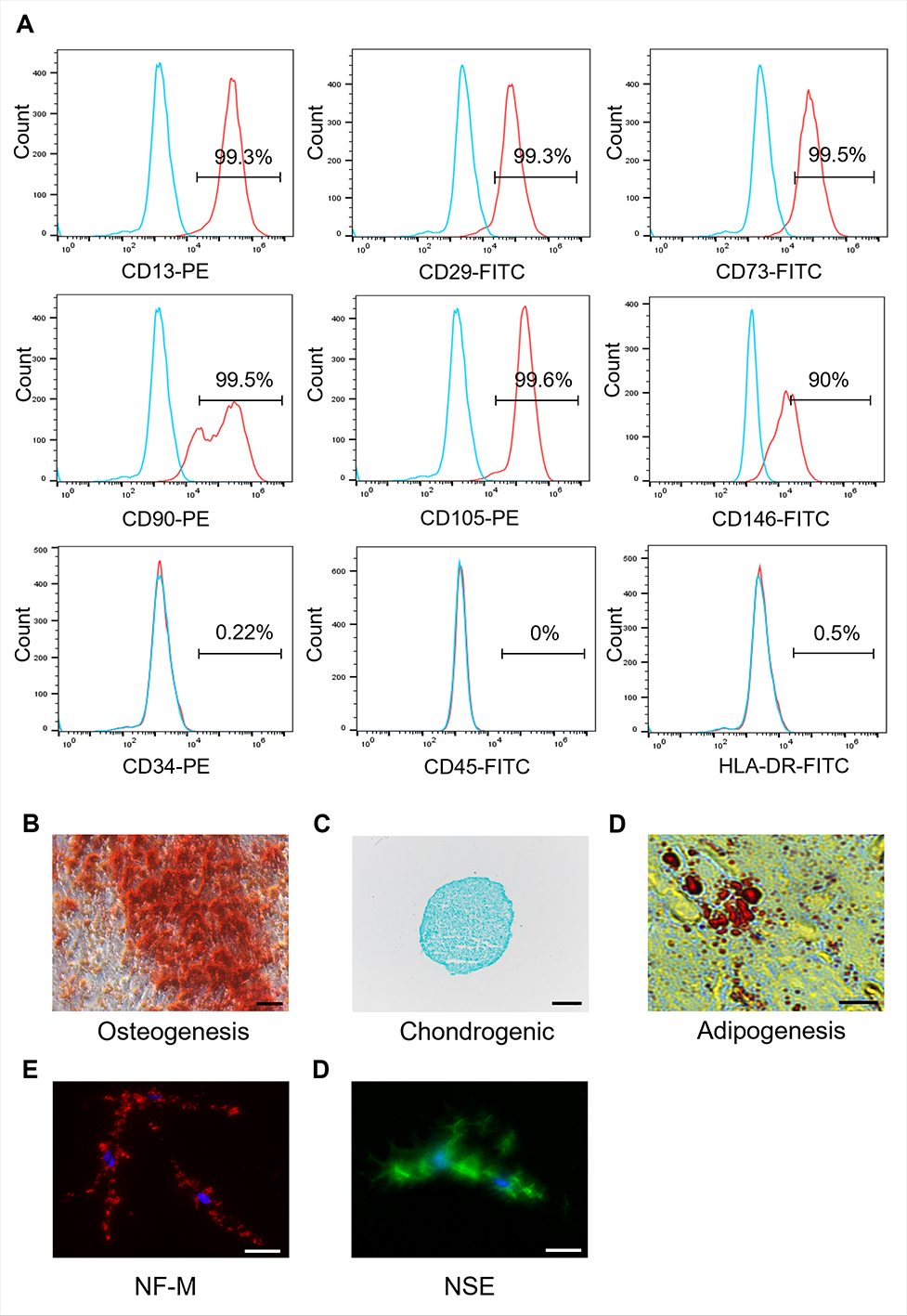


**Figure. S2** **Phenotype of En-PSCs. (A)** Flow cytometry assay detected the surface markers of En-PSCs: positive for CD90, CD13, CD105, CD146, CD29, CD73, and negative for CD34, CD45 and HLA-DR. **(B)** Alizarin red staining identifies the differentiation of En-PSCs towards osteoblasts. Scale bar,100 μm. **(C)** Alizarin blue staining indicates that En-PSCs possess the potential for chondrogenic differentiation. Scale bar, 200 μm. **(D)** Oil red O staining was used to detect the intracellular lipid droplets induced by En-PSCs. Scale bar, 50 μm. NF-M **(E)** and NSE **(F)** immunofluorescence staining confirmed the neural-like differentiation of En-PSCs. Scale bar, 50 μm. Data are expressed as mean ± SD. *P < 0.05, **P < 0.01, ***P < 0.001, ****P < 0.0001, ns, not significant.


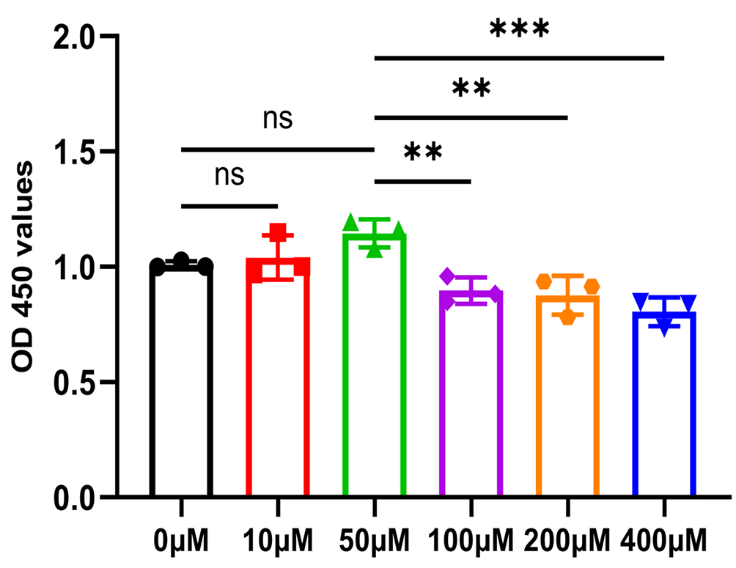


**Fig. S3 Effect of HSYA on the proliferation of En-PSCs detected by a CCK-8 assay.** Proliferative effects of different concentrations of HSYA on En-PSCs.


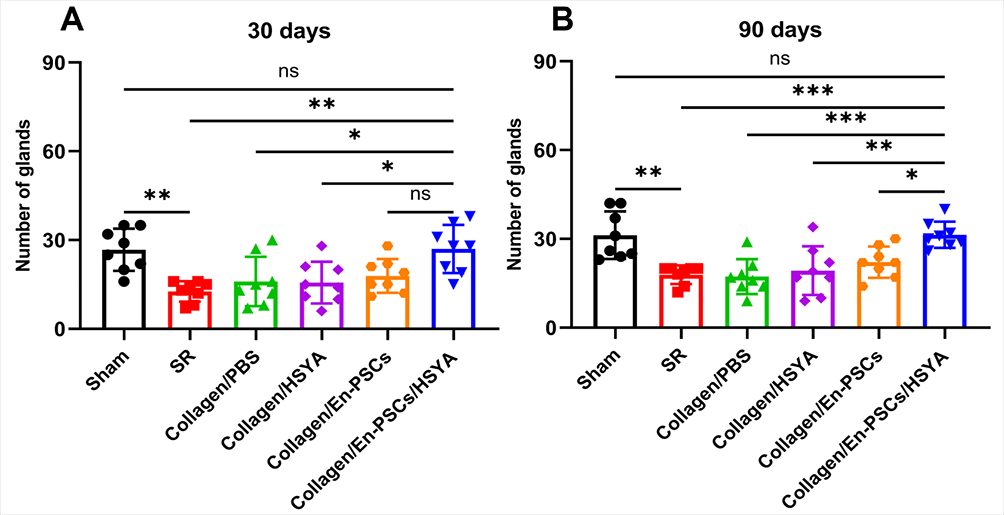


**Fig. S4 Statistical analysis of the number of glands.** The number of glands in the endometrium at 30 days **(A)** and 90 days **(B)** after the operation in each group. The experiment was conducted in triplicate for each group (n = 8 uteri). Data are expressed as mean ± SD. *P < 0.05, **P < 0.01, ***P < 0.001, ****P < 0.0001, ns, not significant.


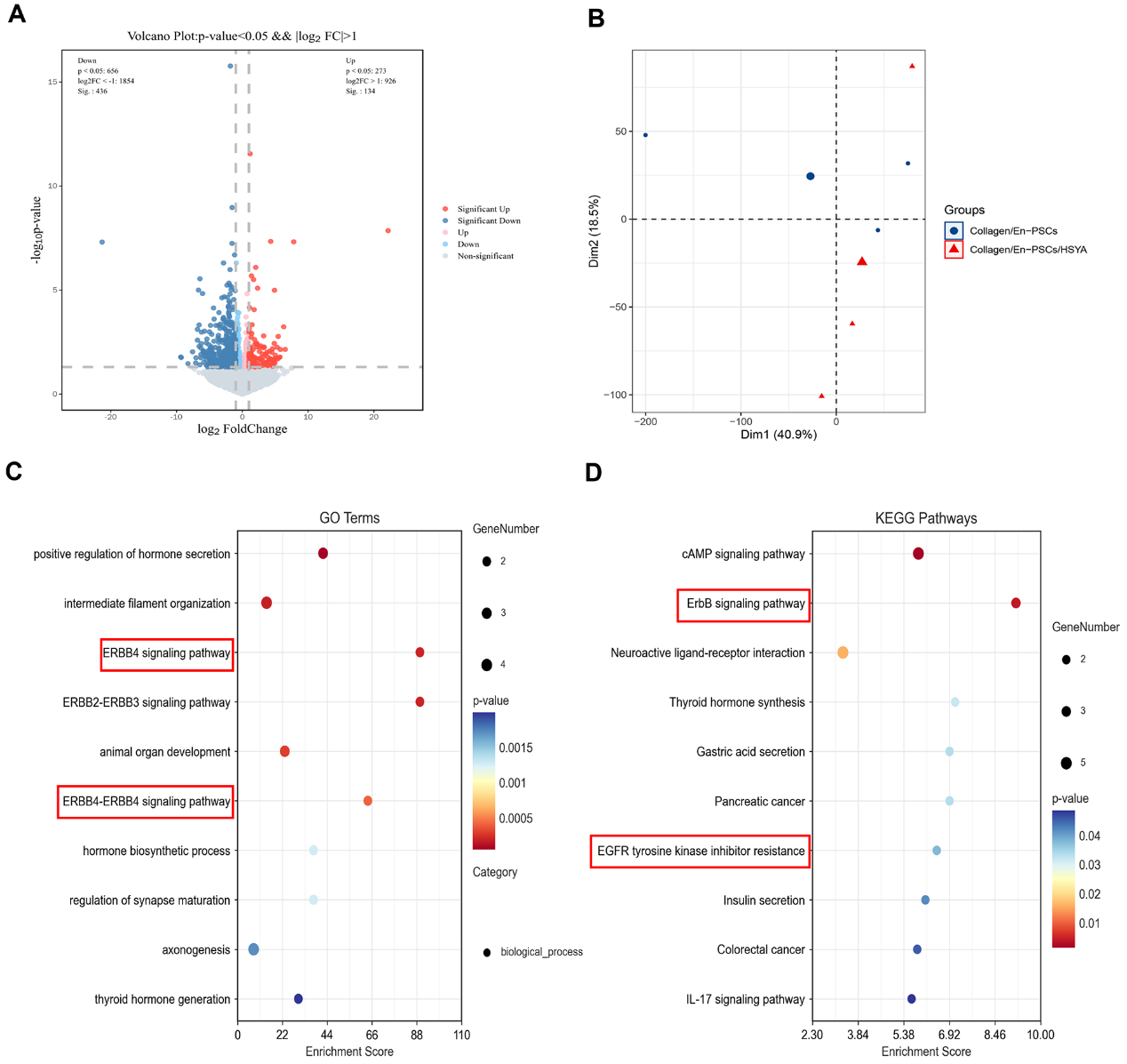


**Figure S5 RNA-seq results suggesting changes in the molecular level of En-PSCs after HSYA treatment. (A)** Volcano plot of genes differentially expressed in En-PSCs before and after HSYA stimulation. The red dots show the upregulated genes, and the blue dots show the downregulated genes. **(B)** Structural map of PCA in the treated and control groups. **(C)** GO enrichment analysis. **(D)** Pathways identified by KEGG pathway enrichment analysis.


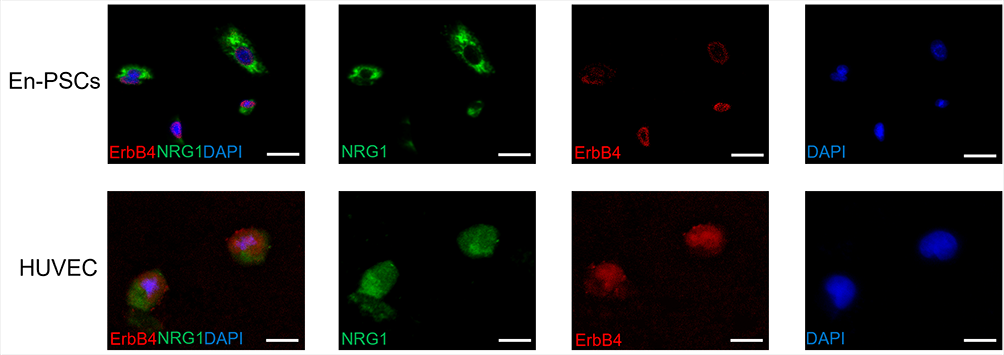


**Fig. S6 Immunofluorescence staining for NRG1/ErbB4.** En-PSCs and HUVECs express both NRG1 and ErbB4. Scale bar 50 μm.


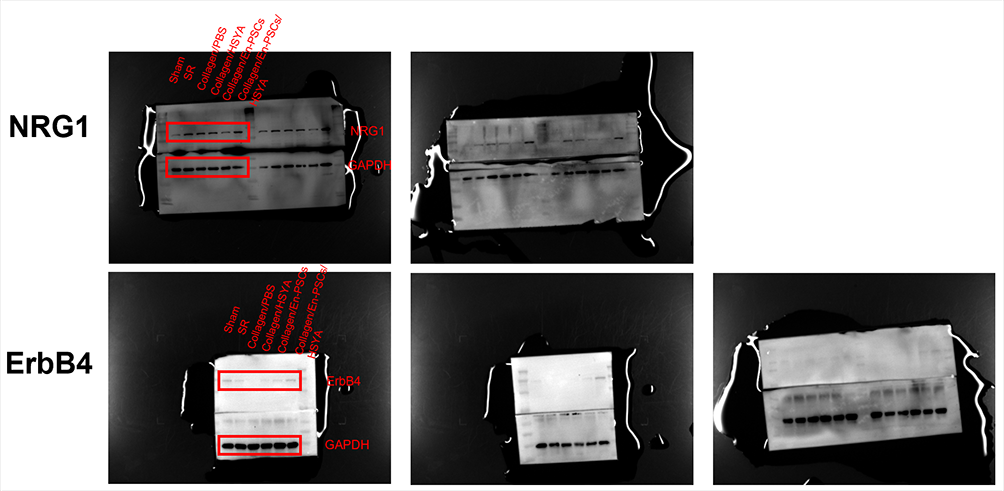


**Fig. S7 Full-length blots.** Full-length blots of NRG1 with ErbB4.
